# Superharmonic microbubble Doppler effect in ultrasound therapy

**DOI:** 10.1088/0031-9155/61/16/6154

**Published:** 2016-07-29

**Authors:** Antonios N Pouliopoulos, James J Choi

**Affiliations:** Noninvasive Surgery and Biopsy laboratory, Bioengineering Department, Imperial College London, London SW7 2AZ, UK; j.choi@imperial.ac.uk

**Keywords:** Doppler effect, therapeutic ultrasound, acoustic cavitation, microbubble dynamics

## Abstract

The introduction of microbubbles in focused ultrasound therapies has enabled a diverse range of non-invasive technologies: sonoporation to deliver drugs into cells, sonothrombolysis to dissolve blood clots, and blood-brain barrier opening to deliver drugs into the brain. Current methods for passively monitoring the microbubble dynamics responsible for these therapeutic effects can identify the cavitation position by passive acoustic mapping and cavitation mode by spectral analysis. Here, we introduce a new feature that can be monitored: microbubble effective velocity. Previous studies have shown that echoes from short imaging pulses had a Doppler shift that was produced by the movement of microbubbles. Therapeutic pulses are longer (>1 000 cycles) and thus produce a larger alteration of microbubble distribution due to primary and secondary acoustic radiation force effects which cannot be monitored using pulse-echo techniques. In our experiments, we captured and analyzed the Doppler shift during long therapeutic pulses using a passive cavitation detector. A population of microbubbles (5  ×  10^4^–5  ×  10^7^ microbubbles ml^−1^) was embedded in a vessel (inner diameter: 4 mm) and sonicated using a 0.5 MHz focused ultrasound transducer (peak-rarefactional pressure: 75–366 kPa, pulse length: 50 000 cycles or 100 ms) within a water tank. Microbubble acoustic emissions were captured with a coaxially aligned 7.5 MHz passive cavitation detector and spectrally analyzed to measure the Doppler shift for multiple harmonics above the 10th harmonic (i.e. superharmonics). A Doppler shift was observed on the order of tens of kHz with respect to the primary superharmonic peak and is due to the axial movement of the microbubbles. The position, amplitude and width of the Doppler peaks depended on the acoustic pressure and the microbubble concentration. Higher pressures increased the effective velocity of the microbubbles up to 3 m s^−1^, prior to the onset of broadband emissions, which is an indicator for high magnitude inertial cavitation. Although the microbubble redistribution was shown to persist for the entire sonication period in dense populations, it was constrained to the first few milliseconds in lower concentrations. In conclusion, superharmonic microbubble Doppler effects can provide a quantitative measure of effective velocities of a sonicated microbubble population and could be used for monitoring ultrasound therapy in real-time.

## 1. Introduction

In 1842 Christian Doppler proposed that a relative motion between an acoustic source and an observer introduces a frequency shift in the waveform detected by the observer. Ever since, the so-called Doppler effect has been used in a variety of applications, ranging from radar technology and astronomy, to satellite communication and medicine. In the context of biomedical ultrasound, Doppler imaging is routinely used for the quantification of blood flow and the diagnosis of pathologies, owing to the spectral broadening or the phase shift of the scattered ultrasound waves (Evans and McDicken 2000). The Doppler effect produced from flowing blood scattering ultrasound can provide information about the fluid velocity and directionality in large vessels. For perfusion studies in the heart or other tissues and organs, ultrasound contrast agents are necessary. In contrast-enhanced ultrasonography, pre-formed systemically administered microbubbles are introduced into the bloodstream and act as ultrasound contrast agents due to their ability to enhance the acoustic signal received from within the vasculature (Stride and Saffari 2003, Cosgrove 2006). Simultaneous measurement of vascular flow and perfusion dynamics is thus possible owing to the presence of microbubbles (Bruce *et al* 2004, Tremblay-Darveau *et al* 2014).

Microbubbles are compressible encapsulated gas particles typically comprised of a lipid or protein shell and a heavy-gas core, with a size range between 1 and 5 *μ*m (Lindner 2004). When sonicated at MHz frequencies, microbubbles respond to the rarefactional and compressional phases of the ultrasonic field by expanding and contracting, a behavior known as acoustic cavitation (Leighton 1994). Microbubbles undergoing acoustic cavitation are subject to primary and secondary acoustic radiation, or Bjerknes, forces (Bjerknes 1906). Primary radiation forces are produced by the interaction of the cavitation nuclei with the primary acoustic field generated by an external source (e.g. an ultrasound transducer). Momentum is transferred from the acoustic wave to the microbubbles, yielding a non-zero net force which depends on the spatial gradient of the acoustic field and the microbubble volumetric oscillation amplitude. The secondary square-law forces are due to inter-bubble coupled interactions and can be either attractive or repulsive, depending on the phase difference in the pulsation of the neighboring microbubbles (Apfel 1988). In the case of a free traveling wave, the direction of the primary Bjerknes force on an individual bubble coincides with the ultrasound propagation direction (Dayton *et al* 1997). Sonication with parameters relevant to medical imaging showed that the magnitude of this force on isolated microbubbles is such that they can achieve instantaneous velocities up to 0.5 m s^−1^ (Dayton *et al* 2002).

Tortoli *et al* were the first to report that microbubble movement due to primary Bjerknes forces resulted in a frequency shift in the detected microbubble emission spectra (Tortoli *et al* 1999a, 1999b). Using a 4 MHz single element transducer that generated 1.3 *µ*s-long sinusoidal pulses at various acoustic pressures, they measured a negative frequency shift on the order of 1 kHz. The frequency shift in the experimental spectra was confirmed by numerical simulations of microbubbles subjected to ultrasound radiation force and drag force from the fluid (Tortoli *et al* 1999b). Modification of parameters like the pulse repetition frequency, the pulse duration and especially the acoustic pressure, led to a different magnitude of asymmetric spectral broadening (Tortoli *et al* 1999a), although always negative with respect to the harmonic peak. Under laminar flow conditions, even though the harmonic peak was shifted due to the fluid flow, the microbubble translation consistently broadened the peak towards the negative frequencies, indicating that the majority of insonified microbubbles, especially those close to resonance, were forced to move away from the receiving transducer (Tortoli *et al* 2000). At acoustic pressures above the microbubble destruction threshold (>500 kPa), microbubble collapse led to a symmetric increase of the broadband emissions without affecting the shape of the harmonic peak, confirming that the asymmetric broadening is due to radiation force effects (Tortoli *et al* 2005). Acoustic streaming was also investigated as a possible cause for the detected frequency distortions, yet it was found that its contribution was negligible at the microsecond time scale (Tortoli *et al* 2001).

All the aforementioned studies were conducted in pulse-echo mode and with imaging pulse shapes and sequences, i.e. at center frequencies within the range 2.5–8 MHz, pulse lengths of up to 10 cycles (or several microseconds) and pulse repetition frequencies on the order of kHz. Hence, the calculated velocities on the order of m s^−1^ were the instantaneous microbubble velocities during the on-time and the detected shifts were attributed to the inter-pulse movement of the cavitation nuclei (Tortoli *et al* 2009). In stark contrast with imaging applications, microbubble-mediated focused ultrasound therapies are typically conducted using low-frequency (<2 MHz) and long-pulse (>1 000 cycles) sonication (Ferrara *et al* 2007, Coussios and Roy 2008). Low-frequency pulses offer the advantages of deeper penetration and larger treatment volumes while long pulse lengths have been shown to increase the induced bioeffect, such as blood-brain barrier opening (Hynynen *et al* 2001, Konofagou 2012) and sonothrombolysis (de Saint Victor *et al* 2014). Although therapeutic ultrasound centre frequencies are typically below the resonance frequency of an average-sized, isolated, and coated microbubble (van der Meer *et al* 2007), the effective resonance frequency of a microbubble population can be within the 0.5–1 MHz range (Yasui *et al* 2009). Long ultrasound pulses displaced microbubbles to a larger extent (Palanchon *et al* 2005). At low pressures, microbubble activity was sustained over long periods (Hitchcock *et al* 2011) while high pressures induced inertial cavitation and rapid microbubble destruction (Chen *et al* 2003, Pouliopoulos *et al* 2014). Microbubbles sonicated with long pulses on the order of milliseconds were found to develop speeds of 1 m s^−1^ during sonication using high-frame rate imaging (Koruk *et al* 2015). The microbubble movement ceased upon contact with the distant wall, yet a continuous redistribution of the microbubble population was observed at high concentrations (Koruk *et al* 2015).

Monitoring of bubbles during ultrasound therapy has been recently demonstrated using ultrasound color Doppler (Zhang *et al* 2015). Color Doppler is an active ultrasound imaging technique, which generates estimates of velocities based on the phase shifts between the echoes of imaging pulses (Evans *et al* 2011). In the aforementioned study, cavitation bubbles spontaneously formed in a high-pressure ultrasound beam (i.e. histotripsy) were correlated with an increase in the color Doppler signal. The increase was attributed to the motion of the surrounding tissue caused by cavitation within the focal area and was used to assess tissue fractionation (Zhang *et al* 2015). This approach is useful for high-pressure therapy but does not provide information about net bubble movement through the field.

A number of methods have been developed to monitor microbubble-mediated acoustic cavitation in low-pressure ultrasound therapy. Using single-element passive cavitation detection (PCD), the duration, type and magnitude can be determined both *in vitro* (Radhakrishnan *et al* 2013, Gruber *et al* 2014, Pouliopoulos *et al* 2014, Shamout *et al* 2015) and *in vivo* (Tung *et al* 2010, O’Reilly and Hynynen 2012, Graham *et al* 2014). By passively capturing the acoustic cavitation signals using a multi-element array and applying suitable beamforming algorithms (Gyöngy and Coussios 2010, Coviello *et al* 2015), one can further localize the acoustic events in space in order to assess the spatiotemporal distribution of acoustic cavitation activity *in vitro* (Choi and Coussios 2012) or to monitor the *in vivo* therapeutic bioeffects in real-time (Choi *et al* 2014). All the aforementioned monitoring techniques have been hitherto limited to quantifying the duration, type, magnitude and location of microbubble acoustic activity within the focal area. Currently it is not feasible to deduce the sonicated microbubble translational dynamics based on the generated acoustic emissions; hence a method of quantifying effective velocities of contrast agents during ultrasound therapy is required.

In this work we evaluated the presence and magnitude of the microbubble Doppler shift due to the long-pulse and low-frequency ultrasound exposure of microbubbles. We hypothesized that the spectral broadening observed in imaging sequences also occurs with therapeutic pulse shapes and sequences. To identify discernible effects, we studied the microbubble acoustic emissions above the 10th harmonic (hereafter referred to as ‘superharmonics’). Quantification of the microbubble axial translational dynamics based on the Doppler effect may be useful in real-time therapy monitoring applications by adding the extra dimension of calculating microbubble velocities during ultrasound treatment.

## 2. Methods

### 2.1. Experimental setup

In-house manufactured microbubbles were prepared following previously described methods (Pouliopoulos *et al* 2014, Shamout *et al* 2015). We measured the microbubble characteristics using optical microscopy and an automated counting algorithm (Sennoga *et al* 2010), finding an average size of 1.2  ±  0.8 *μ*m and a diameter range of 0.5–8.9 *μ*m. The microbubble solution was diluted in phosphate buffer saline (PBS) to a concentration of 5  ×  10^7^ microbubbles ml^−1^. Most measurements were conducted using this nominal concentration. To quantify the effect of microbubble concentrations, initial solutions were also diluted in PBS to a concentration of 5  ×  10^6^, 5  ×  10^5^, and 5  ×  10^4^ microbubbles ml^−1^.

**Figure 1.**
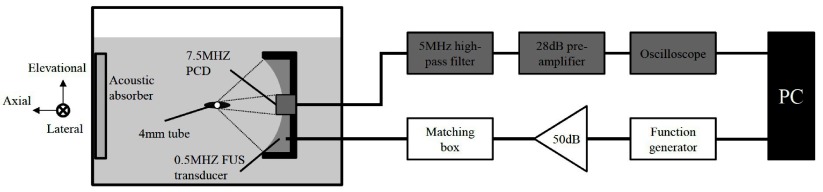
Experimental setup with the emission (white) and detection (grey) circuits. A 4 mm diameter tube filled with microbubbles was submerged in a water tank and sonicated with a 0.5 MHz focused ultrasound (FUS) transducer. The microbubble acoustic emissions were captured with an inserted and co-aligned 7.5 MHz passive cavitation detector (PCD), recorded with an oscilloscope and saved to a PC for off-line processing.

Microbubbles were infused and left to set in a 4 mm in diameter vessel-like elastomer tube (Saint-Gobain Performance Plastics, Paris, France) which was fixed in a tank containing deionized and degassed water (figure 1). Microbubbles were not made to flow during sonication, in an effort to minimize the spectral distortions due to the fluid flow. An elastomer tube was used instead of a plexiglas tube because the latter was shown to produce Doppler artefacts due to longitudinal and shear waves (Tortoli *et al* 1997). An arbitrary waveform generator (33500B Series, Agilent technologies, Santa Clara, CA, USA) produced the therapeutic pulses (pulse length: 100 ms or 50 000 cycles; center frequency: 0.5 MHz), which were amplified using a 50 dB RF amplifier (Precision Acoustics Ltd, Dorchester, UK), and applied to the focused ultrasound transducer through an impedance matching box (Sonic Concepts Inc., Bothell, WA, USA). Focused ultrasound was emitted using a 0.5 MHz focused ultrasound transducer (part number: H107, diameter: 64 mm, lateral full width at half maximum (FWHM) at the focus: 5.85 mm, focal length: 62.6 mm, axial FWHM: 29.78 mm; Sonic Concepts Inc., Bothell, WA, USA), whose focus coincided axially and elevationally with the tube. We selected the acoustic pressures (peak-rarefactional pressure (PRP): 75, 147, 217, 294, and 366 kPa) based on previous studies with the same microbubble formulation (Pouliopoulos *et al* 2014) to produce cavitation modes ranging from non-inertial to inertial cavitation.

Microbubble acoustic emissions were detected with an inserted 7.5 MHz focused ultrasound transducer (part number: U8423589, diameter: 12.7 mm, focal length: 60 mm; Olympus Industrial, Essex, UK) operating in PCD mode. The two transducers were coaxially aligned and their foci overlapped. A 0.2 mm polyvinylidene fluoride (PVDF) needle hydrophone (Precision Acoustics Ltd, Dorchester, UK) was used to determine the foci prior to the experiments. Hydrophone measurements did not reveal waveform distortions caused by nonlinear propagation. A 5 MHz analogue high-pass filter (part number: MH-500 P-C-P; Allen Avionics, Mineola, NY, USA) filtered out the fundamental frequency and low frequency reflections from the tube. The filtered signal was amplified with a 28 dB pre-amplifier (Stanford Research Systems, Sunnyvale, CA, USA) and then recorded with a digital oscilloscope (sampling rate 25 MSa s^−1^; Tektronix, Bracknell, UK). Emission and detection processes were controlled with Matlab (The Mathworks, Natick, MA, USA) and all data were saved to a PC for off-line processing.

### 2.2. Signal processing

**Figure 2.**
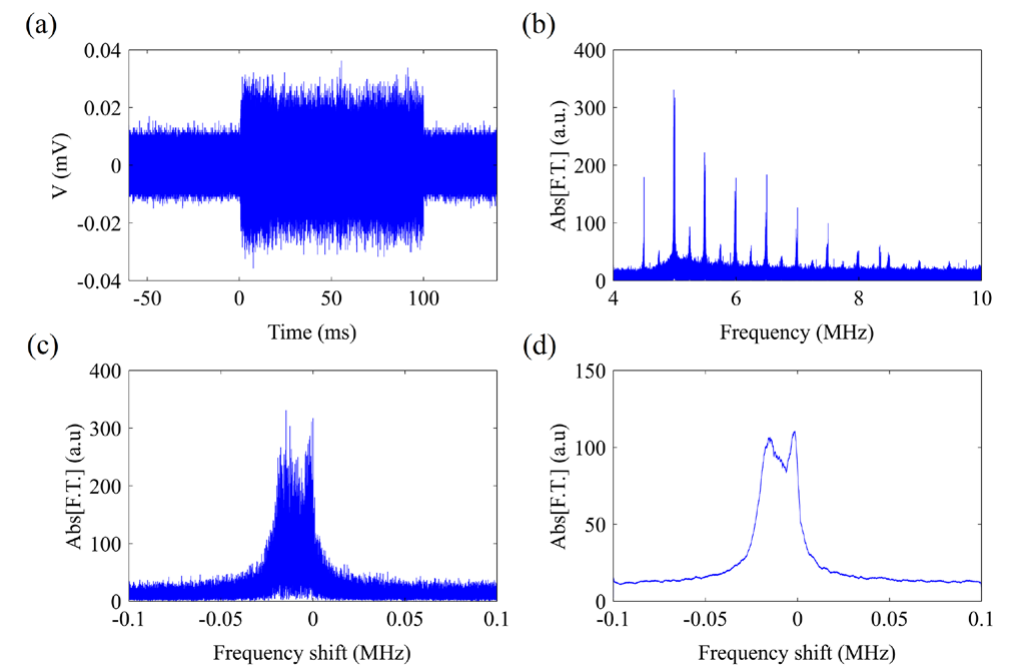
Signal processing algorithm. Acoustic emissions from microbubbles exposed to 0.5 MHz ultrasound were captured as a (a) time-domain signal that was then (b) spectrally analysed for either the entire sonication duration or within segments of time. (c) Superharmonics to the driving centre frequency were windowed and then (d) smoothed using a moving average filter. The superharmonic shown in (c) and (d) is the 10th harmonic from (b).

Raw data were processed in Matlab (figure 2). We spectrally analyzed the received emissions (figure 2(a)) either for the entire sonication duration (i.e. 100 ms) or within specified time windows. In contrast to previous studies on the Doppler shift, which were concerned with its presence in ultrasound imaging (Tortoli *et al* 1999a, 1999b, 2000, 2001), our study had a number of differences that were unique to ultrasound therapy. The first difference is that therapeutic ultrasound exposure of microbubbles involves longer pulse lengths and lower centre frequencies. Longer pulse lengths (>1 ms) have the microbubbles experience a primary acoustic radiation force for a longer duration, thereby increasing average velocities. Lower centre frequencies (<2 MHz), when compared at the same pressures to imaging frequencies (>2 MHz), cause larger microbubble responses (e.g. acoustic cavitation, primary and secondary acoustic radiation force) (Holland and Apfel 1990). The second difference lies in the method whereby acoustic emissions from the microbubbles are captured and stems from the use of therapeutic parameters. In imaging, the same transducers are typically used in pulse-echo mode. In therapy, acoustic emissions are captured while the pulse, due to its length, is still being emitted from the transducer. As a result, a second transducer, called a passive cavitation detector, is used so that the driving signal does not interfere with the received acoustic emissions. The final difference is the method of acoustic emissions analysis. In imaging, the first or second harmonic are typically analysed while in therapy, the greater microbubble response and longer pulse lengths allow for superharmonics to be examined. Doppler shifts increase with the frequency analysed which makes the quantification and characterisation of velocities easier.

A fast Fourier transform over the entire signal duration was obtained (figure 2(b)) and each of the superharmonics was isolated, with the spectrum being segmented up to 0.1 MHz on both sides of the expected superharmonic peak (figure 2(c)). The baseline spectra were not subtracted in the Fourier domain since the tube’s acoustic reflections were negligible in the superharmonic regions. Due to the noisy data, we applied a moving average filter with different span in order to smooth the spectrum (figure 2(d)). A frequency span of 3 kHz for the smoothing process resulted in discernible peaks of lower amplitude without significant distortion of the spectral fine structure. To elucidate the time-resolved frequency content of the microbubble acoustic emissions, we calculated the spectrograms with a 1 ms-long Hanning window and a 0.5 ms overlap between adjacent segments.

To derive effective microbubble velocities we used the Doppler equation, modified due to the fact that microbubbles are simultaneously both receivers and emitters of sound. Thus, a factor of 2 was added to account for the ‘double’ Doppler effect (Tortoli *et al* 2000):

1 \begin{eqnarray*}{{v}_{\text{eff}}}=\frac{c}{2\,{{f}_{\text{sh}}}}\,\Delta f.\end{eqnarray*}

where $\Delta \,f$ is the frequency shift, ${{f}_{\text{sh}}}$ is the frequency of the superharmonic, ${{v}_{\text{eff}}}$ is the effective microbubble velocity and $c$ is the speed of sound.

Every measurement was repeated multiple times using the same experimental conditions (i.e. *n*  =  5 for the pressure dependence and *n*  =  3 for the concentration dependence experiments), thus the spectra presented in this study are averaged across the repetitions (i.e. different sonications) in the frequency domain. All measurements are presented as mean  ±  standard deviation.

## 3. Results

### 3.1. Detection of the microbubble superharmonic Doppler peak

**Figure 3.**
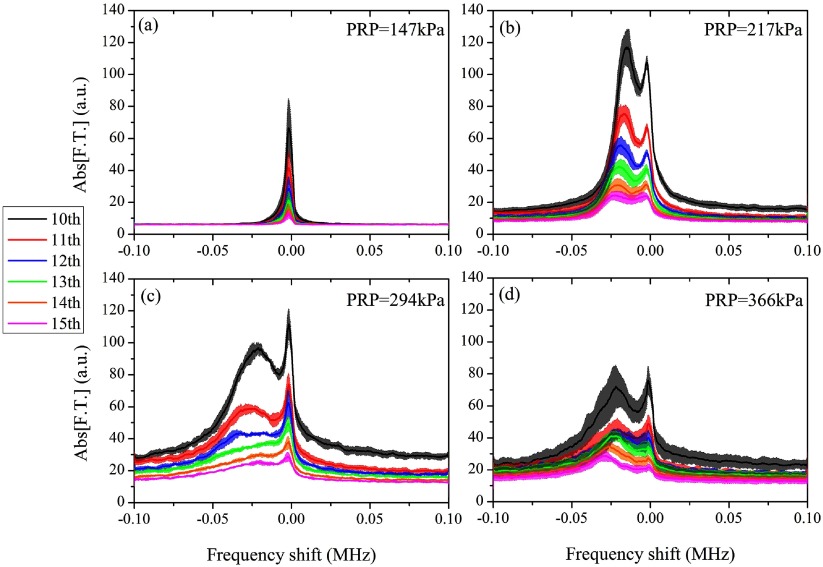
Doppler shift across the superharmonics for PRP equal to (a) 147 kPa, (b) 217 kPa, (c) 294 kPa and (d) 366 kPa (microbubble concentration: 5  ×  10^7^ ml^−1^). Solid lines and shaded areas represent average spectra and one standard deviation respectively (*n*  =  5).

The aim of this study was to detect and characterize the superharmonic microbubble Doppler effect under low-frequency and long-pulse therapeutic sonication. By passively recording the acoustic emissions of the sonicated microbubbles, we were able to isolate and analyze superharmonic regions for further analysis. In our specific experiments, we analyzed the 10th to 15th harmonic (figure 3). The spectra presented here were averaged in the Fourier domain across repetitions and the shaded areas represent one standard deviation. At low pressures (PRP: 75 kPa) harmonic signals were not detected due to the low signal-to-noise ratio at higher harmonics. At moderate pressures (PRP: 147 kPa), a subtle broadening of the superharmonic peak was observed with a negative shift bias (figure 3(a)). Increasing the peak negative pressure above 200 kPa led to the emergence of a secondary peak adjacent to the primary harmonic peak (figure 3(b)). Despite the gradual decrease in the amplitude of higher-order superharmonics the structure of the spectral broadening was consistent across the frequencies. We attributed the asymmetric broadening of the superharmonic peak to the microbubbles moving away from the PCD transducer, thus we will refer to the secondary peak as the ‘Doppler peak’. At sonication pressures higher than 217 kPa, the detected Doppler peak decreased in amplitude and generally increased in width (figures 3(c), (d) and 5).

**Figure 4.**
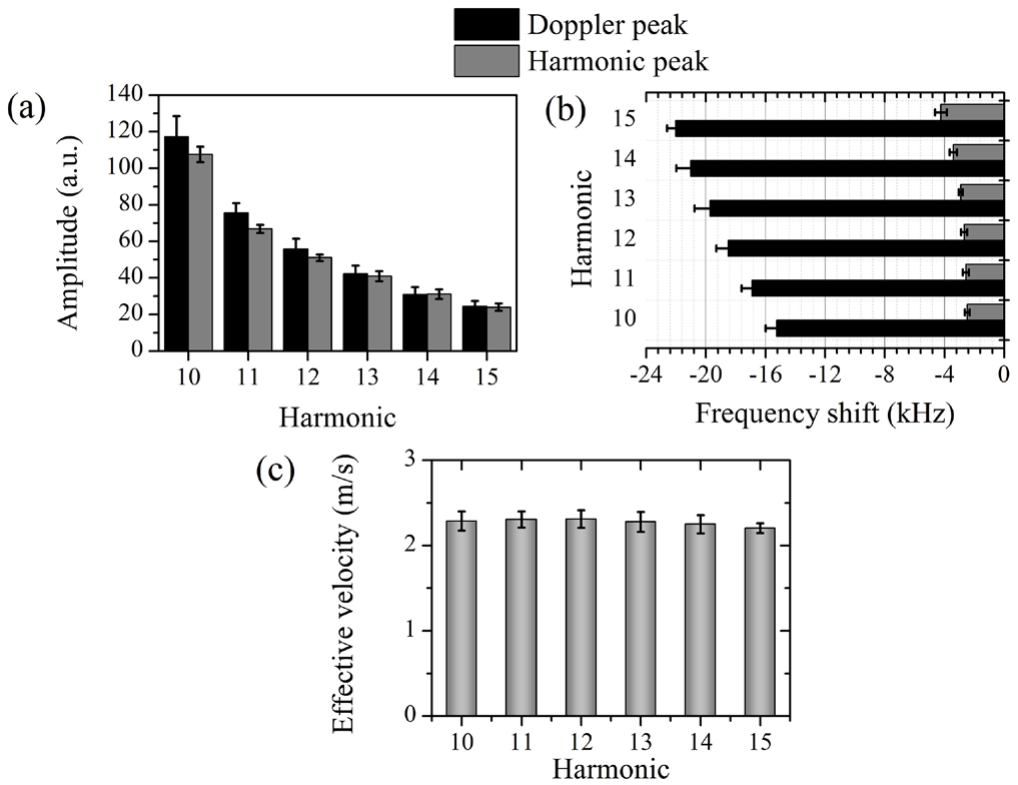
Doppler shift quantification across the harmonics (PRP: 217 kPa, microbubble concentration: 5  ×  10^7^ ml^−1^). (a) Doppler and harmonic peak amplitude. (b) Frequency shift of the Doppler and the harmonic peak. (c) Estimated effective velocities across the superharmonics. All measurements are averages across the repetitions and are presented as mean  ±  S.D (*n*  =  5).

**Figure 5.**
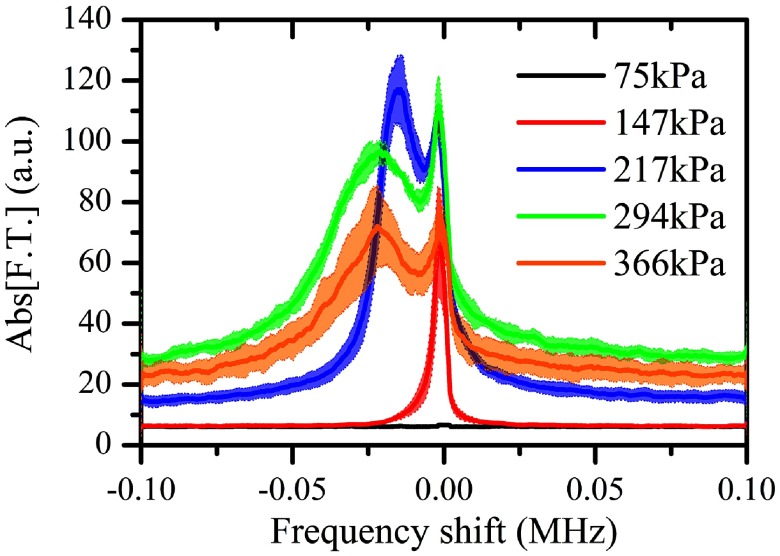
Microbubble superharmonic Doppler shifts varied with the peak rarefactional pressure (10th harmonic, microbubble concentration: 5  ×  10^7^ ml^−1^). Solid lines and shaded areas represent average spectra and one standard deviation respectively (*n*  =  5).

### 3.2. Doppler peak characterization across the superharmonics and the acoustic pressures

We characterized the superharmonics and their neighboring Doppler peaks for 217 kPa PRP as an illustrative example (figure 4). The detected amplitude of both peaks decreased for higher harmonics due to a lower emitted amplitude and higher attenuation. In this example, the amplitude of the Doppler peak was higher than the superharmonic peak (figure 4(a)). At higher acoustic pressures the ratio between the Doppler and superharmonic peak amplitude was generally lower than 1 (figure 3). Using equation (1), we converted the detected negative shifts (figure 4(b)) to effective microbubble velocities during sonication (figure 4(c)). Effective velocities characterized the polydisperse microbubble population translational dynamics and were computed as a comparative measurement across the tested conditions. Our measurements showed that despite the differences in the frequency shifts (figure 4(b)), the estimated velocities are within the same range for all the superharmonics (figure 4(c)). At a PRP of 147 kPa, the calculated effective microbubble velocity, averaged across the harmonics, was found to be 2.26  ±  0.03 m s^−1^ (*n*  =  6). Interestingly, the primary harmonic peaks were also moderately shifted with respect to their expected positions (figure 4(b)).

There were a number of qualitative differences in the spectral broadening across the acoustic pressures (figure 5). Higher pressures produced a larger frequency shift of the Doppler peak. The width of the Doppler peak increased with pressure until 300 kPa. The amplitude of the distinct Doppler peak decreased with pressure for PRP higher than 217 kPa. The noise floor increased with pressure, with the exception of the highest tested pressure (PRP: 366 kPa).

**Figure 6.**
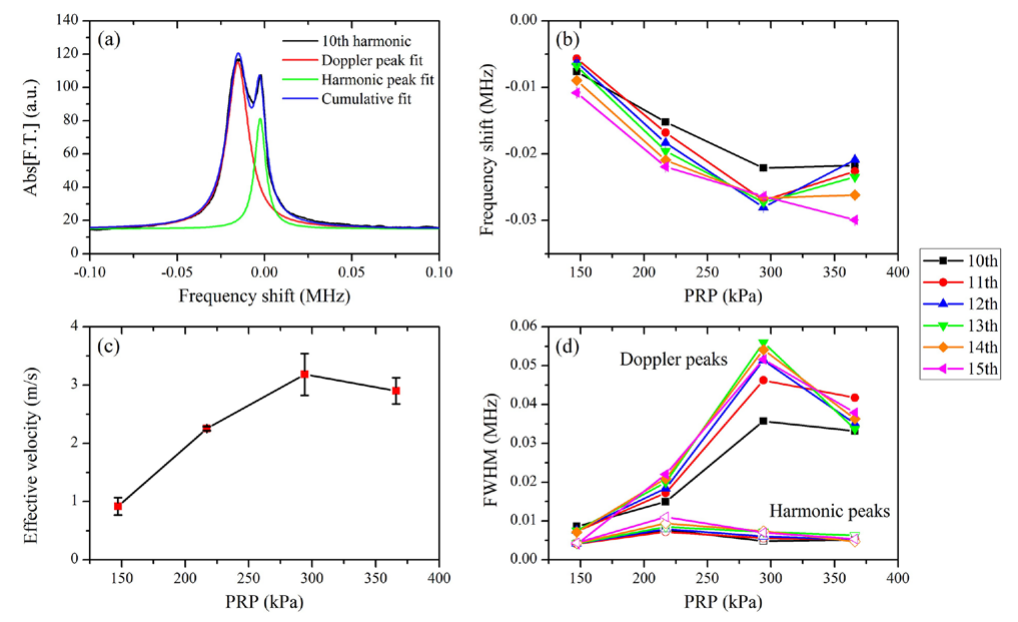
Quantification of Doppler shift dependence on acoustic pressure. (a) Lorentzian double-peak fitting gave the optimal fitting accuracy (10th harmonic, PRP: 217 kPa). (b) Frequency shifts of the Doppler peak over the acoustic pressure across the superharmonics. (c) Estimated effective velocities of the sonicated microbubbles at different acoustic pressures. Averaging was conducted across the harmonics (*n*  =  6). (d) FWHM for the Doppler and the harmonic peaks.

To quantify the aforementioned qualitative observations, we fitted the two detected peaks with Lorentzian functions (figure 6(a)). We chose Lorentzian peaks over Gaussian peaks, due to the better fitting accuracy with the former function. All Lorentzian fits provided high fitting accuracy (*R*^2^  >  0.9). Fitting results showed that the Doppler peak frequency shift decreased linearly with pressure, ranging from  −5 kHz to  −30 kHz (figure 6(b)). This linear decrease was not followed by several of the superharmonics at the highest pressure, which was likely due to inaccurate fitting and large standard deviations of the experimental spectra (figure 3(d)). Using equation (1), we converted these frequency shifts into effective velocities (figure 6(c)). Although indiscernible, the secondary peak present at PRP of 147 kPa yielded an effective microbubble velocity of 1 m s^−1^. For higher acoustic pressures we estimated faster movement of the microbubble population, reaching up to 3.1 m s^−1^ for PRP  =  294 kPa. Another interesting observation was that the full width at half maximum (FWHM) of the Doppler peak generally increased with pressure, while the primary harmonic peak FWHM was effectively unaltered (figure 6(d)). The discrepancy at the highest pressure was also evident at the Doppler peak FHWM measurement. We have discarded the standard deviations from figures 6(b) and (d) for visibility. The standard deviations in figure 6(b) were on the order of 1 kHz, which was similar to those shown in figure 4(b). The variability on the FWHM of each superharmonic at different pressures can be estimated in figure 3.

**Figure 7.**
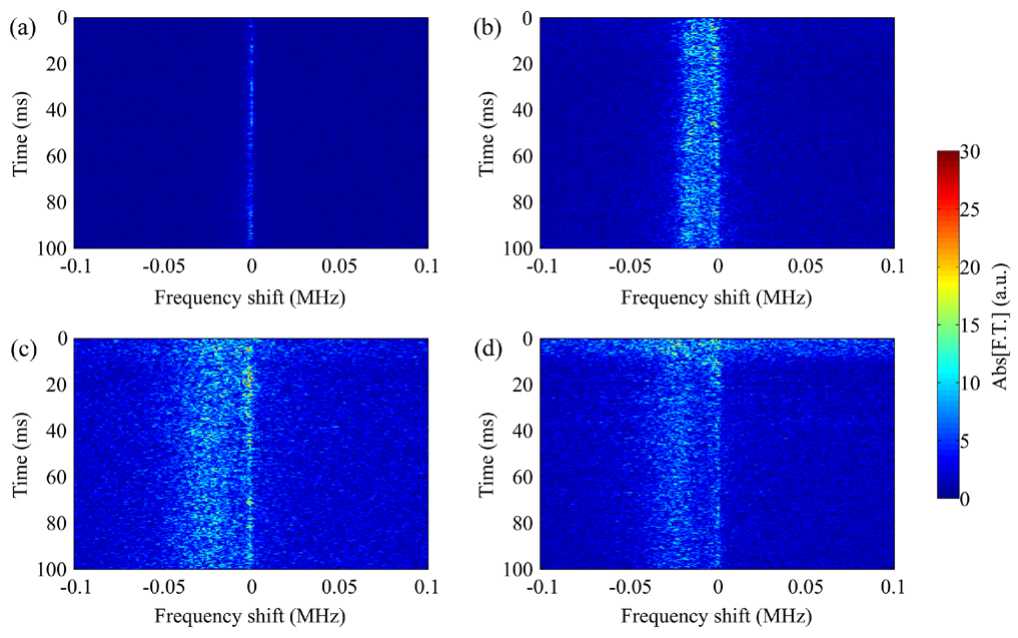
Time-resolved Doppler shift for sonication at different peak rarefactional pressures. (a) 147 kPa, (b) 217 kPa, (c) 294 kPa, and (d) 366 kPa (10th harmonic, microbubble concentration: 5  ×  10^7^ ml^−1^).

To understand the temporal evolution of the frequency shift at different acoustic pressures, we temporally analyzed the microbubble acoustic emissions during sonication (figure 7). At low acoustic pressures (figure 7(a)) the Doppler peak was not separated from the primary harmonic peak as shown for the Fourier transform performed throughout the sonication period (figures 3(a) and 5). However, the moderate negative shift appearing as a ‘tail’ to the primary superharmonic persisted during the entire sonication (figure 7(a)). By increasing the peak rarefactional pressure, the spectral broadening was evident throughout the sonication duration and was continuously on the order of tens of kHz (figures 7(b) and (c)). In the case of sonication at 366 kPa, increased broadband signal was detected in the first 10 ms of the ultrasound exposure, followed by a Doppler shift of lower amplitude (figure 7(d)).

### 3.3. Effect of microbubble concentration on the detected Doppler shifts

**Figure 8.**
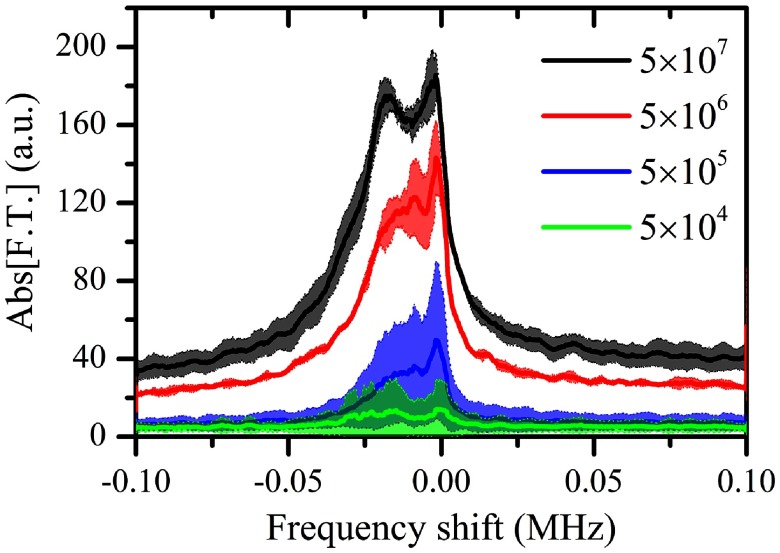
Superharmonic Doppler shifts for different microbubble concentrations (10th harmonic, PRP: 217 kPa). Solid lines represent average spectra while the shaded areas denote one standard deviation (*n*  =  3).

Due to the dense microbubble populations that were used, the effects of the secondary acoustic radiation forces were not expected to be insignificant. We were interested in studying the effect of microbubble concentration on the produced spectral broadening, hence we reduced the tested concentration in 10  ×  steps and repeated the experiments at a PRP of 217 kPa (*n*  =  3). By reducing the number of sonicated microbubbles, we obtained a spectrum of progressively lower amplitude, as expected (figure 8). The signal-to-noise ratio yielded with the lowest concentration (5  ×  10^4^ microbubbles ml^−1^) was too low to allow for meaningful interpretation. For comparison, the clinically recommended dose of Definity^®^ contrast agents in ultrasound imaging is 2  ×  10^6^ microbubbles ml^−1^ (Unger *et al* 2004). The frequency shift of the Doppler peak decreased for lower concentrations (table 1), indicating lower effective velocities for the dilute samples. Although the standard deviations were large for the lower concentrations (figure 8), the FWHM of the Doppler peak generally decreased with concentration (table 1).

**Table 1.** Quantification of the superharmonic Doppler effect for different microbubble concentrations (10th harmonic, PRP: 217 kPa).

Concentration (microbubbles ml^−1^)	Frequency shift (MHz)	Doppler peak FWHM (MHz)	Effective velocity (m s^−1^)
5 × 10^7^	−0.017	0.027	2.55
5 × 10^6^	−0.014	0.026	2.1
5 × 10^5^	−0.013	0.021	1.95

**Figure 9.**
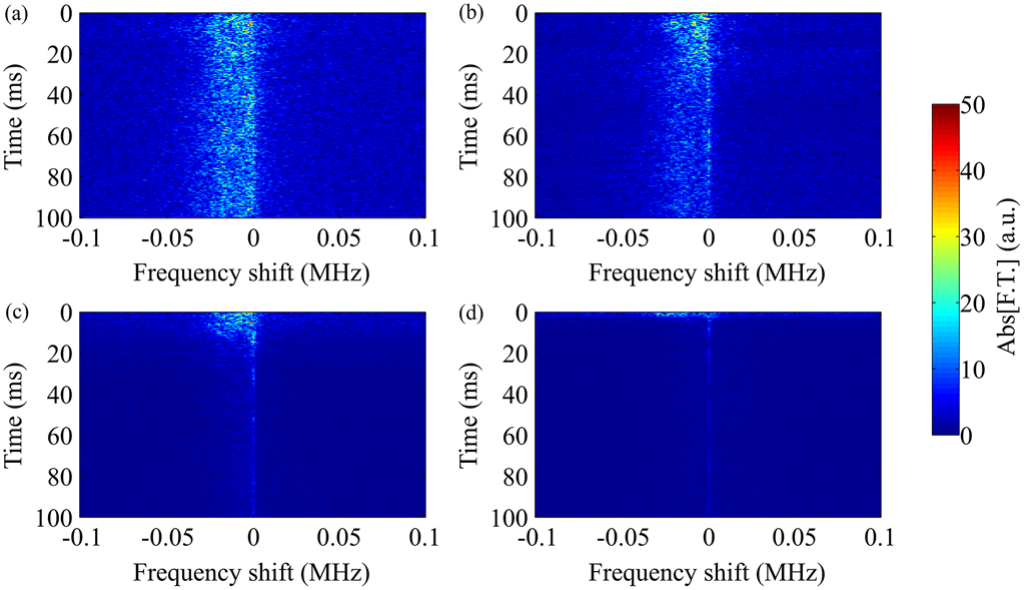
Time-resolved Doppler shift for different microbubble concentrations. (a) 5  ×  10^7^, (b) 5  ×  10^6^, (c) 5  ×  10^5^, and (d) 5  ×  10^4^ microbubbles ml^−1^ (10th harmonic, PRP: 217 kPa).

A time-resolved frequency analysis of the acoustic emissions revealed the reasons for these differences. Although the frequency shift persisted for the entire sonication period in the dense population case (figure 9(a)), spectrograms showed that it is limited mainly within the first milliseconds of the 100 ms long pulse (figures 9(b)–(d)). For a concentration of 5  ×  10^6^ microbubbles ml^−1^, the shift on the order of tens kHz persisted for approximately 25 ms, while for the concentration of 5  ×  10^5^ microbubbles ml^−1^ the duration was 10 ms. In terms of signal amplitude, we observed a gradual decrease in the microbubble response possibly due to microbubble destruction or translation out of the focal volume of the PCD transducer.

## 4. Discussion

### 4.1. The origin of microbubble Doppler effect in the therapeutic regime

We have demonstrated that low-frequency and long-pulse sonication of microbubbles, which is routinely used in ultrasound therapy, produces an asymmetric broadening of the superharmonic acoustic emissions. A secondary peak was shown to emerge near the harmonic peak, negatively shifted up to  −30 kHz with respect to the expected position of the peak (figures 3, 5, 8). A striking difference between the imaging and therapeutic regimes is the scale of the Doppler shift. Whereas in all previous studies the frequency shift was on the order of hundreds of Hz (Tortoli *et al* 1999a, 1999b, 2000, 2005), in this study the detected Doppler peaks were shifted tens of kHz away from the harmonic peak. Another distinct observation from previous studies is that their observed Doppler effect appeared as a broadening of the harmonic peak (Tortoli *et al* 2000, 2005) while we observed a distinct peak that can be quantified and characterized. The reasons are that previous studies examined the fundamental frequency and that the shifts were not due to continuous translation of the acoustic sources, but due to the inter-pulse microbubble movement during the off-time of the sequence (Tortoli *et al* 2009). Hence, whereas the instantaneous m s^−1^ velocities should translate into tens of kHz of frequency shift, the detected broadening was constrained to a few hundreds of Hz (Tortoli *et al* 2005, 2009).

Time-resolved spectral analysis showed that there are different kinetic behaviors during sonication (figures 7 and 9). For large concentrations we observed a continuously negative frequency shift which contradicts the notion of a unidirectional movement of the microbubbles. Assuming a velocity on the order of m s^−1^ (figures 4(c), 6(c) and table 1), a bubble initially near the proximal wall of the tube would reach the distal wall in a few milliseconds. However, the continuous shift (figures 7 and 9(a)) indicates another mechanism which may last for the entire sonication period. A possible explanation is streaming produced by microbubbles displacing fluid in the direction of ultrasound propagation. This hypothesis is based on recently published experimental observations (Cho *et al* 2015, Koruk *et al* 2015). Koruk *et al* showed that in high concentrations microbubbles can continuously deform an elastic interface for tens of milliseconds (Koruk *et al* 2015). Microbubbles appeared to be trapped within vortices in the focal volume, thus continuously redistributing during sonication. In our experimental setup, such vortices may contribute to the persistence of spectral broadening. The focal volume of the 7.5 MHz PCD transducer is smaller than that of the 0.5 MHz focused ultrasound transducer. Hence, the receiving volume was embedded within the  −3 dB beamwidth of the sonicating transducer. It is thus likely that the captured signals originate from the microbubbles moving away from the detector, while spinning in the vortex loop and entering the PCD focal volume in a recurring manner. The detected frequency shifts were not likely to be caused by large microbubble clusters spinning around their centre-of-mass due to strong inner attractive forces, since such movement should introduce a symmetrically positive shift in the acoustic emissions. For low concentrations, microbubbles have been optically shown to move along the axial direction in m s^−1^ velocities (Koruk *et al* 2015), however the microbubble velocity was not quantified. Our results for low concentrations (table 1 and figure 9) are in accordance with this optical evidence, since we observed that the Doppler shift is present only in the first few milliseconds for concentrations below 5  ×  10^6^ microbubbles ml^−1^ (figures 9(b)–(d)). With fewer microbubbles, the creation of vortex loops was less likely to occur, since fluid would be displaced at a lower extent. Also, lower microbubble concentrations reduce or eliminate the formation of bubble clusters or the coalescence to form larger bubbles (Fan *et al* 2014, Bader *et al* 2015), which could reduce the amplitude of the acoustic emissions.

A separate study showed that microbubbles sonicated at center frequencies between 1 and 2.25 MHz caused fluid streaming velocities on the order of cm s^−1^ and triggered symmetric vortices at the focal volume outskirts (Cho *et al* 2015). It was argued that the fluid streaming patterns were produced by bubble clustering (Koda *et al* 2011) or coalescence (Postema *et al* 2004) and the collective microbubble translation. Microbubble clustering in a capillary has been shown to occur within hundreds of milliseconds or even seconds (Kotopoulis and Postema 2010). However, the effective resonance frequency of the microbubble population decreases at high concentrations (Yasui *et al* 2009) thus faster microbubble dynamics may emerge at low-frequency therapeutic sonication. A possible mechanism that may influence the detected acoustic emissions is microbubble coalescence (Postema *et al* 2004). Long-pulse sonication has been shown to cause fast fusion of the cavitation nuclei into large bubbles (Fan *et al* 2014, Bader *et al* 2015), with radii closer to the resonance size at 0.5 MHz. These large nuclei are likely to produce a large portion of the detected shifts, since the proximity to resonance triggers larger acoustic emissions and primary acoustic radiation forces. Finally, the collective oscillatory behavior of the insonified microbubble population may lead to unexpected resonant behaviors due to the many-body coupled interactions present in such systems (Zeravcic *et al* 2011), thus increasing the individual cavitation nuclei velocities to the m s^−1^ regime. Microbubbles closer to resonance undergo larger radial oscillations, experience larger primary radiation forces and thus move faster. Hence, we speculate that the physical basis of the Doppler peak shape and width is dependent on the microbubble size distribution and sonication frequency.

Despite the underpinning complexity of the microbubble dynamics leading to the emergence of the secondary Doppler peak, the linear increase in the frequency shift across the superharmonics allowed improved accuracy in the calculated effective velocities, especially at moderate pressures (figures 4(c) and 6(c)). At higher pressures, the Doppler peak broadened and shifted further away from the primary superharmonic peak (figures 3, 5, 6), therefore the calculated effective velocities progressively increased (figure 6(c)). At the highest tested pressure this trend was inverted, most likely due to increased microbubble destruction rates (figure 7). Although for PRP lower than 200 kPa there is no detectable broadband signal, for pressures above the inertial cavitation threshold (Choi and Coussios 2012, Pouliopoulos *et al* 2014) the noise floor rises (figures 7(c) and (d)). Inertial cavitation and rapid microbubble collapse can be inferred from the high levels of broadband signal in the beginning of high-pressure sonication (figure 7(d)). Combining the spectral content of the first milliseconds with the frequency shifts of the remaining sonication results in the narrower, less intense and less shifted Doppler peak produced by the full-duration fast Fourier transform (figure 5), which in turn affects the effective velocity estimation (figure 6(c)).

Similar Doppler shifts were observed in the ultraharmonics (odd multiples of half the fundamental frequency) apart from the superharmonics (data not shown). Although small in magnitude thus less discernible, ultraharmonics are produced only by microbubbles undergoing nonlinear oscillations (Lauterborn 1976, Apfel 1997), hence the asymmetric frequency broadening has to be attributed only to the microbubble redistribution. We have excluded non-linear ultrasound propagation and reflections from the tube or the tank as causes of the detected shift, since such effects are not expected to appear in the superharmonic range.

### 4.2. Clinical importance

Our strategy of identifying and quantifying the microbubble translational dynamics through the Doppler effect may find application in a variety of therapeutic applications. The advantage of the superharmonic Doppler effect is that it can provide information about the therapeutic outcome of microbubble-mediated ultrasound therapies in a passive, thus non-invasive and safe manner. Real-time estimation of the effective microbubble velocities (figures 4(c) and 6(c)) would allow for increased control of the induced bioeffect. Microbubbles need to move towards a surface or vessel wall to induce desired bioeffects in applications such as clot dissolution (Acconcia *et al* 2013, 2014, 2015, Bader *et al* 2015), intracellular drug delivery (Fan *et al* 2014, Shamout *et al* 2015), acoustic particle palpation (Koruk *et al* 2015), and molecular imaging or therapy (Dayton *et al* 1999). Passive monitoring of the Doppler effect through analysis of superharmonic microbubble emissions allows users to track microbubble velocities using standard passive cavitation detection systems. Thus, our method provides an inexpensive, easily accessible, and readily available tool for the estimation of microbubble movement near surfaces or vessel walls. Currently, microbubble-seeded acoustic cavitation activity can be passively monitored *in vivo* in terms of its type, magnitude, and duration, using passive cavitation detection (Tung *et al* 2010, O’Reilly and Hynynen 2012, Graham *et al* 2014), or its position, using passive acoustic mapping (Choi *et al* 2014). Yet these techniques are not able to quantify the microbubble translation due to primary and secondary acoustic radiation forces. Our proposed method introduces the dimension of acoustic cavitation translational dynamics within the sonicated area. Generally, a thorough understanding of the microbubble dynamics is expected to result in safer and more efficient microbubble-mediated focused ultrasound therapies, which can be simultaneously monitored using the developed technique.

In addition to clinical applications, the proposed method can be used to understand the translational dynamics of sonicated microbubbles. It may also be used in other applications where moving particles are present, such as in non-destructive testing, surface cleaning, etc.

### 4.3. Limitations of the study

We have shown that the acoustic signature of translating microbubbles can be used to quantify the population translational dynamics during sonication. The reported effect is expected to be prominent mainly in large vessels, due to the large number of bubbles within the sonicated population and the wide space available for the microbubble dynamics to unravel. Large vessels like arteries and veins have high flow rates, yet in this work we decided to study the microbubble dynamics in static conditions. As a result, the detected peaks are expected to present an additional negative or positive shift *in vivo*, depending on the direction of the blood flow (Tortoli *et al* 2000). Also, our microbubble solution was diluted in PBS and not in blood. This may affect the microbubble dynamics *in vivo*, since the properties of blood are different from PBS (e.g. viscosity), hence the generated acoustic cavitation mode or magnitude may alter (Apfel and Holland 1991). Finally, the calculated effective velocities are derived from the frequency shift of the Doppler peak and are thus estimates of the average microbubble velocity.

## 5. Conclusions

In conclusion, we detected and characterized the Doppler effect yielded under low-frequency and long-pulse ultrasound exposure of translating microbubbles. Analysis of the superharmonics revealed a secondary peak negatively shifted a few tens of kHz with respect to the primary harmonic peak. The position, amplitude, and width of this Doppler peak were found to depend on the acoustic pressure and the microbubble concentration. Using our signal processing algorithm we measured effective microbubble velocities on the order of m s^−1^. Superharmonic microbubble Doppler effects can be used for the real-time monitoring of the cavitation nuclei axial velocities during microbubble-mediated focused ultrasound therapies. In our future work, we aim to incorporate physiologically relevant flow and perform simultaneous high-frame rate optical observations to confirm the validity of the acoustically estimated velocities.
